# A Case Report of Neurosarcoidosis Presenting as a Lymphoma Mimic

**DOI:** 10.1155/2016/7464587

**Published:** 2016-10-12

**Authors:** Gurcharanjeet Kaur, Lauren Cameron, Olga Syritsyna, Patricia Coyle, Agnes Kowalska

**Affiliations:** Department of Neurology, Stony Brook University Hospital, Stony Brook, NY, USA

## Abstract

*Objective.* To describe a unique presentation of neurosarcoidosis.* Background.* Central nervous system involvement is rare in sarcoidosis. Sarcoidosis can be severe and can be mistaken for systemic lymphoma.* Case Description.* A 55-year-old right-handed white male with past medical history of obstructive sleep apnea, Raynaud's disease, and Hashimoto's thyroiditis was noted to have cognitive decline over a duration of few weeks and 20 lb weight loss. His neurologic exam (including cranial nerves) was normal except for five-minute recall. Head CT revealed a lacrimal gland mass, confirmed on brain MRI, which was suspicious for lymphoma on brain PET/MRI. Subsequent whole-body FDG PET/CT scan showed multiple enlarged lymph nodes. Bone marrow biopsy was negative. Serum and CSF ACE levels were within normal limits. Supraclavicular lymph node biopsy before steroids therapy was initiated and revealed multiple noncaseating granulomas, diagnostic of “sarcoidosis.” He was treated with daily prednisone for two months, followed by weekly infliximab. Brain MRI two months after treatment with prednisone showed decrease in size of lacrimal lesion, and brain PET/MRI showed normal brain metabolism pattern after five months. Neurocognitive evaluation three months after diagnosis demonstrated improvements in memory abilities.* Discussion.* Both clinically and radiographically, neurosarcoidosis can mimic systemic lymphoma. Biopsy in these types of cases is necessary to establish the diagnosis.

## 1. Introduction

Sarcoidosis is a multisystem disease which involves formation of inflammatory lesions known as granulomas. Central nervous system's involvement is rare. Clinical neurologic complications occur in approximately 5% of patients [1–3]. Signs of neurologic involvement are usually seen in patients with florid systemic sarcoidosis.

Diagnostic criteria for neurosarcoidosis in the absence of central nervous system (CNS) histology are not firmly established. A clinically compatible picture, exclusion of other neurological diseases, and histological confirmation of disease elsewhere are generally required [4].

We present a case report of neurosarcoidosis presenting as a lymphoma mimic.

## 2. Case Report 

**Figure 1:**
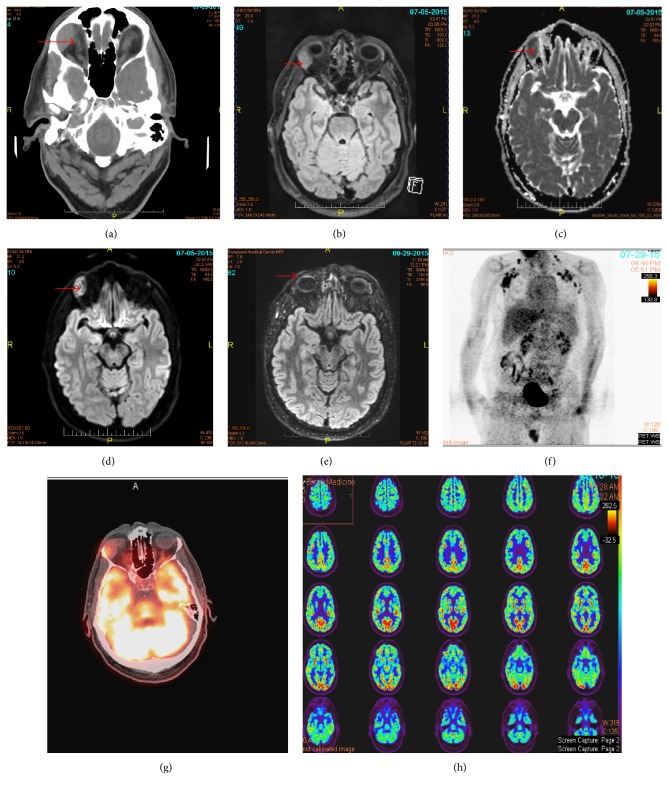
Head CT revealing a lacrimal gland mass (a). Lacrimal gland mass on initial Axial FLAIR (b). Restricted diffusion in the lacrimal glass mass (c and d). Repeat MRI after initiation of prednisone revealing decreased size of lacrimal lesion (e). Hypermetabolic foci corresponding to lymph nodes in bilateral neck, axillary regions, chest, pelvis, and inguinal regions on whole-body PET/CT (f). Prominent hypermetabolic lesion in the right lateral orbit before prednisone (g). No evidence of hypermetabolic lesions or regional hypometabolism within the brain on PET/MRI 5 months after prednisone and infliximab (h).

A 55-year-old right-handed white male with past medical history of obstructive sleep apnea, Raynaud's disease, and Hashimoto's thyroiditis presented to the emergency department with a several weeks' history of acute cognitive decline. He had verbal repetitions, anxiety, and forgetfulness. Review of systems revealed loss of appetite with 20 lb weight loss over three months. Neurologic consultation was requested for the cognitive decline. Neurology examination (including cranial nerves) was unremarkable except for five-minute recall. Emergent testing included EEG, CT of the head, brain MRI, and brain PET/MRI. Head CT revealed a lacrimal gland mass (Figure 1(a)). Brain MRI with contrast revealed an enhancing right lacrimal gland mass measuring up to 2.65 cm with restricted diffusion suspicious for lymphoma in addition to chronic small vessel disease (Figures 1(b), 1(c), and 1(d)). Additional outpatient work-up included whole-body FDG PET/CT scan, lumbar puncture, paraneoplastic panel, and neurocognitive testing. Cognitive testing revealed intact functioning across most domains of cognition; however, in contrast to these areas of intact functioning, he was densely amnestic with marked impairment in problem-solving abilities. Lumbar puncture results were normal except for elevated opening pressure (26.5 cm of H_2_O). ACE in both serum and CSF was normal. Mayo clinic serum paraneoplastic panel had negative result. Brain PET/MRI showed decreased radiotracer accumulation in the bilateral temporal lobe and frontal lobes, compatible with Frontotemporal Dementia. Subsequent body PET/CT scan showed prominent hypermetabolic lesion in the right lateral orbit and lymph nodes in bilateral neck, axillary regions, chest, pelvis, and inguinal regions, most consistent with lymphoma (Figures 1(f) and 1(g)). Aspirate bone marrow biopsy was negative for lymphoma. Supraclavicular lymph node biopsy before steroid therapy was initiated and revealed multiple noncaseating granulomas, diagnostic of sarcoidosis. The patient was treated with daily prednisone for two months, followed by weekly infliximab anti-tumor necrosis factor-*α* (anti-TNF-*α*) infusion.

A repeat brain MRI with contrast done at one and two months after initiation of steroids showed marked decrease in size of the right lacrimal lesion to about 10 × 13 mm in the axial plane (Figure 1(e)). Chest CT without contrast two months after initiation of prednisone showed marked improvement in his supraclavicular, axillary, and mediastinal adenopathy with a residual 1.2 cm right axillary lymph node. Brain PET/MRI five months after steroid and infliximab treatment showed normal brain metabolism pattern (Figure 1(h)). Neurocognitive evaluation three months after diagnosis demonstrated improvements in memory abilities.

## 3. Discussion

There is a wide variety of clinical presentations of neurosarcoidosis. Both clinically and radiographically, neurosarcoidosis can be difficult to diagnose. There are few case reports of neurosarcoidosis presenting as an acute infarct with restricted diffusion on MRI [5]. There is no known cure for neurosarcoidosis. Immunosuppression is the primary means of controlling the disease, and corticosteroids are the cornerstone of therapy. Treatment options are limited; however, there is more evidence suggesting that steroids and immunomodulatory agents such as infliximab may improve clinical outcomes, which may be due to the anti-TNF-*α* effect on reducing oxidative stress [6, 7]. There is evidence that TNF-*α* regulates synaptic transmission in the brain and that this cytokine can produce spatial memory impairment in mice [8]. Anti-TNF-*α* treatment is beneficial in reducing cognitive failure, as well as disease activity and fatigue [9]. More recently, Moravan and Segal [10] demonstrated that combination treatment with mycophenolate mofetil and infliximab is a promising therapeutic approach for refractory neurosarcoidosis. The data for infliximab are not robust; however, case reports have proven that it has some benefit in patients with neurosarcoidosis [11].

## 4. Conclusion

Our patient did not have lymphoma and had a good response to corticosteroids and infliximab. However, the clinical presentation was suspicious for lymphoma. Often, FDG PET/CT scan can be misleading and may appear to be neoplastic rather than inflammatory. ACE levels in both CSF and serum are not always positive. Biopsy in these cases is necessary to establish correct diagnosis.
